# Perceptions of drug use within a UK Bengali community

**DOI:** 10.4103/0019-5545.42397

**Published:** 2008

**Authors:** Mohammad Shams Uddin, Dinesh Bhugra, Mark R. D. Johnson

**Affiliations:** The Crisis Resolution and Home Treatment Service-Counties, OSL House, East Link, Meridian Business Park, Leicester, LE19 1XU, UK; 1 Health Service and Population Research Department, Institute of Psychiatry, King's College London, Box PO25, De Crespigny Park, London SE5 8AF, UK; 2 Professor, Health and Social Care, Mary Seacole Research Centre, De Montfort University, Charles Fears Campus, 266 London Road, Leicester LE2 1RQ, UK

**Keywords:** Asians, drugs, ethnic minority, substance use

## Abstract

**Aim:**

The study set out to explore the perceptions and knowledge of drug use of the Bangladeshi origin population in Leicester through local Mosques and community and resource centers for recruiting subjects.

**Setting and Design:**

A triangulated methodology was used for this research. A review of all available literature was carried out to establish if there was evidence of a drug problem among the Bangladeshis in the UK along with questionnaires and interviews.

**Materials and Methods:**

A triangulated methodology was used for this research. A review of all available literature was carried out along with questionnaires as well as semi-structured interviews using self-designed questionnaires.

**Results and Conclusions:**

Only 66 questionnaires (16.5%) were returned (46 males and 20 females). These and qualitative exploratory interviews with a small number of community leaders confirmed that drug-related problems exist among the Bangladeshi community, especially in the younger age group and are recognized as such but help seeking is often problematic. An understanding of the perceptions of the Bangladeshi population is useful in developing culturally appropriate services for this group.

## INTRODUCTION

The availability and use of illegal drugs and resulting addictions presents a serious problem in the UK. Illegal drugs are widely available and causing serious social and health problems.[1–3] The consequences of such drug use include serious and organized crime perpetrated by drug dealers and addicts as well as harm to individual users of substances.

Evidence suggests that drug use is on the increase among the South Asian population in the UK, especially the Bangladeshi communities.[4,5] This problem is not publicly acknowledged by the Bangladeshi community because stigma affects the status of the family in society[6] and the notion of shame in the family is very strong.

Setting in Leicestershire: The geographical catchment area has a population 615,000 and nearly half of the population live in market towns, or urban areas close to Leicester.[7] The County has a long history of settlement from other parts of the world. In the early 1970s, there was significant migration to Leicestershire of people of Bangladeshi and Gujarati origin. More recently, there has been movement out of the city of Leicester into the more rural areas. The vast majority of Bangladeshis in the UK as well as in this region come from the rural Sylhet district in Bangladesh. They speak various dialects of the national language (Bangla) and the first generation of migrants at least had a low level of literacy. In respect of religion, the majority are Sunni Muslims.[7]

The qualifications of older Bangladeshi men and women in the UK have been found to be extremely low, and young Bangladeshi people also remain the least well qualified of all the UK minority ethnic groups. There is nevertheless a high penetration of this group in British economy and society through the operation of catering establishments and yet there is low residential ownership compared with British Indian and Pakistani groups indicating a different profile.

### Literature review:

Electronic and manual literature searches were employed to gather information on the current pattern of drug use among the Bangladeshis in UK. Databases were searched without any restrictions and inclusive of all the publication years to produce the largest number of hits. Gray literature items (a document such as a newspaper, a popular magazine article, or a local study, which has not been published in a peer-reviewed journal) were also included in this review. In addition, various search engines were used on the Internet to locate literature.

The inclusion criteria for the literature were deliberately set widely initially to ensure that all relevant literature was included for the review. Therefore, any piece of academic or scientific literature, whether journal article, book chapter, report, or document discussing drug-related issues among the South Asian population in the UK, and especially those relating to Bangladeshis and drugs, Islam and drugs, and young Asians and drugs, was selected for review. Exclusion criteria were related to relevance (including only the UK-based studies) and the use of detailed descriptions which indicated inclusion of the specific population “at risk”, as well as ensuring that purely journalistic or impressionistic reports without reference to reproducible evidence were excluded. These results are illustrated in Tables 1 and 2.

**Table 1:** Other sources of information

Center for Ethnicity and Health (Uni. C. Lancashire)	Fifteen documents via the Internet and post
www.nafas.org (London)	Two documents via Internet
Drug Action Team Leicester	Three government documents
Home Office Publications London	Drugs catalog and 20 documents obtained
www.QED.emcedda.org	Links to over 30 different Internet sites and databases
www.crescentlife.com	Ten articles on Islamic Philosophy and drug addiction

**Table 2:** Search terms and results

Search terms source	Asians and drugs	Asians and illicit substances	Ethnic minority and drug use	Ethnic minority and illicit substances	Illicit substances	Substance use and ethnicity
Academic search elite	*T* = 17	*T* = 0	*T* = 11	*T* = 0	*T* = 32	*T* = 62
	*R* = 0	*R* = 0	*R* = 2	*R* = 0	*R* = 0	*R* = 0
ASSIA + BHI	*T* = 1	*T* = 0	*T* = 6	*T* = 0	*T* = 20	*T* = 19
	*R* = 0	*R* = 0	*R* = 2	*R* = 0	*R* = 0	*R* = 5
BNI (RCN journal's database)	*T* = 0	*T* = 0	*T* = 0	*T* = 0	*T* = 0	*T* = 0
	*R* = 0	*R* = 0	*R* = 0	*R* = 0	*R* = 0	*R* = 0
BMJ database	*T* = 228	*T* = 1153	*T* = 2012	*T* = 253	*T* = 5	*T* = 87
	*R* = 0	*R* = 2	*R* = 0	*R* = 0	*R* = 0	*R* = 0
Cochrane library	*T* = 11	*T* = 0	*T* = 2	*T* = 0	*T* = 15	*T* = 17
	*R* = 0	*R* = 0	*R* = 0	*R* = 0	*R* = 2	*R* = 1
CINAHL	*T* = 34	*T* = 0	*T* = 7	*T* = 0	*T* = 0	*T* = 0
	*R* = 0	*R* = 0	*R* = 0	*R* = 0	*R* = 0	*R* = 0
Expanded academic	*T* = 15	*T* = 600 +	*T* = 8	*T* = 400	*T* = 4	*T* = 222
	*R* = 1	*R* = 3	*R* = 0	*R* = 0	*R* = 0	*R* = 0

***Key**: T* = Total number of hits per search term; *R* = Total number of hits considered relevant for the review.

Only a few studies have examined the prevalence of drug use among the South Asian population in the UK.[8,9] However, even fewer have examined this specifically among the Bangladeshis. In many “comparative” studies with Asian samples,[8] the Bangladeshi, Indian and Pakistani groups are (erroneously) classed as one homogeneous group (despite significant variations in culture, language, religion, and socioeconomic profiles), creating difficulties in ascertaining the level of problems.

In a study by Rogers *et al.*,[10] 33% of Bangladeshi parents reported placing strong restrictions on their children's social activities compared to only 7% of parents from the three other South Asian groups. In another study by Karlsen *et al.*,[11] semi-structured interviews were carried out with a total of 132 adolescents aged 12-13 years. Of these, 34 were Bangladeshis (19 M: 15 F) and the rest from other ethnic groups. A follow-up interview was completed 17 months later. A total of 46% of Bangladeshis (16) could name at least one drug at the first interview and 89% (31) could name one drug 17 months later. This study confirmed that restrictions by their parents did not prevent youngsters from learning about drugs from their peers.

In some parts of the country, the heroin abuse problem is reported to be at an all time high among the Bangladeshis. The London Borough of Tower Hamlets is considered to be the heroin capital of Britain.[12] In that area, Bangladeshis constituted over 30% (*n* = 50,000) of the population in 2000. It was estimated that half of those were under the age of 15. Six years on and in view of the collective evidence to date, the current figure will probably far exceed that reported in 2000. There are reports that young Bangladeshi girls are being targeted, hooked onto drugs, and lured into prostitution to fund their drug habit.[2,13,14]

Carey[13] also reported that heroin was the drug of choice among the Bangladeshi youth on the Ocean Estate of Tower Hamlets, East London. Furthermore, one drug agency reported having 40% Bengali clients on their caseload compared to 10% 5 years ago and that Bengali clients were considerably younger than the other clients.[15]

Chauhan[16] conducted a local needs assessment in Leicester and found that in a sample of 50 participants, Hindu (17), Muslim (13), and Sikh (19), that eight had used cannabis and two had used cocaine in the Muslim sample. Out of all users, 33 (49.2%) said that they had used cannabis, and out of the 33 users, 28 (84.8%) were males. All heroin users were Sikh males. As the focus of that study was drug problems among different religious groups, the national-ethnic origin of the sample was not given, hence it is not known whether any Bangladeshis were in the sample. These were self-reported levels of drug use and the sample was small.

In a study by White,[17] 21 Bangladeshi heroin users were compared with a sample of white heroin users. The study compared Bangladeshi heroin users, drug-using characteristics, and social environment to white users of the same age and sex. The results showed that the Bangladeshi sample started using drugs earlier in life than the white sample, which suggest that restriction in childhood did not appear to work. They were also using larger quantities of stronger drugs. The mean age of first cannabis use among the Bangladeshis was 14.1 years compared to 16.6 (*P* = 0.01) in the white group. The mean age of first heroin use was 17.2 years compared to 20.8 in the white group (*P* = 0.03). Nine out of 21 Bangladeshis (42.8%, *P* = 0.00) in that sample had used heroin before leaving school in comparison to none in the white comparison group.

The Black and minority ethnic (BME) Forum[18] carried out an assessment of need in Kensington, Chelsea, and Westminster. A total of 50 semi-structured questionnaires were administered to the Bangladeshi sample. They found that 38% (19) had used or were using heroin and all heroin users smoked instead of injecting. A further 10% (5) used or were using crack although only 2% (1) of females had used heroin. The method of recruitment of subjects was not clear. The low incidence of females reporting drug use is consistent with findings by White[17] and Fountain *et al.*[5]

When local BME groups were trained to conduct their own needs assessments, in relation to drugs education, prevention, and treatment services, data were gathered from over 12,000 people from 30 ethnic groups in 47 geographical locations across England.[19,20] However, a number of methodologic inconsistencies exist from one assessment to the next, thus preventing the making of any generalizations.

Fountain *et al.*[5] carried out a comprehensive literature review on the pattern of drug use among minority ethnic groups in the UK and reported that heroin was the first drug of choice for Bangladeshi and Pakistani males in some areas.

Thus, it would appear that there is indeed a recognizable drug problem among male Bangladeshis in the UK. The older generation Bangladeshis denies such a problem exists fearing embarrassment (*Sharam* - a word meaning “shame” but having a stronger impact than its English equivalent), stigma, and social exclusion by the community. It seems that where a problem exists, people prefer to keep it hidden and try to deal with it within the family. This is done in an effort to save the family name and honor (*Izzat*). The impact of such denial, fear, and desperation to keep such problems hidden is to prevent those who are affected by drug problems from coming out and seeking help.

## RESPONDENTS AND METHODS

A triangulated approach which used literature review, self-administered questionnaires, and one-to-one semi-structured interviews. The use of postal and self-completion written questionnaires among South Asian populations is known to be problematic, but was felt to be one pragmatic option to attempt to collect quantitative information. Advice was sought from various local communities and academic agencies and professionals while devising the data collection tools and these individuals were involved in the study design.

Utilizing religious institutions is an effective way of approaching hard to reach or vulnerable sections of a community,[21–23] although it will exclude those who are less religious, we feel that this should be the initial approach.

Based on Denscombe's[24] estimates for the return rate for questionnaires, a total of 260, using convenience sampling, were distributed between two Mosques, which were considered to have the largest numbers of Bangladeshis attending for prayers. Snowballing technique was also used to distribute a further 140 questionnaires via youth/community centers and personal contacts.

The questionnaires were piloted with a small number of people to ensure they met the requirements of the study and had face-to-face content validity.

It was decided to use questionnaires written in English, which would be accessible to the target group of younger people as this is the age group most at risk of starting drug use, although this meant that the view of elders may not be possible. Older people from this community are also unlikely to take part in paper-based surveys.

The inclusion criterion for the survey was all self-identified Bangladeshi/British Bengali people, males and females of all ages. The exclusion criterion was anyone not self-identifying as a Bengali or of Bangladeshi origin.

## RESULTS

A total of 66 (16.5%) usable questionnaires were returned and age and gender of respondents recorded. Basic sociodemographic details are shown in Table 3.

**Table 3:** Age/gender of respondents

Age range	Males	Females
Below 16	6	5
17-25	14	8
26-35	10	2
36-45	9	1
46-55	7	2
55 and above	0	2
Total	46	20

The respondents did not differ from non-respondents. A total of 21 males and seven females felt that there was a problem of drug use in the community, while the rest either said ‘No’ or ‘Don't know’ or ‘prefer not to say’ [Table 4].

**Table 4:** Do you know any Bangladeshis currently using drugs?

	Males	Females
Yes	21	7
No	8	4
Don't know	2	3
Prefer not to say	15	6
Total	46	20

For those that responded ‘yes’, the main reasons highlighted were that youngsters were socializing more and teenagers were copying their friends. Many felt that drugs were easily available at schools and work and that ‘everyone was doing it.’ Peer pressure and young people mixing in with the wrong crowd at schools were factors highlighted by both male and female respondents.

The majority of respondents highlighted cannabis (ganja, marijuana, weed, and spliff) as the main drug of choice followed by heroin, cocaine, and speed. Respondents also commented that youngsters usually start with stealing cigarettes from home and this casual smoking very quickly leads to cannabis and other drug use.

Twenty-nine males (63%) felt that drugs problems affected males only and three felt that it affected both males and females while 11 females (55%) felt that the problems were with males and two felt that it was with females only observations. Males only were more likely to report that (37, 55.87% vs. 15, 22.65%) drug problems were confined to the younger population (age range 17-25), which is also consistent with the findings from the interviews. The majority of the respondents (51, 77.01%) felt that drug problems most commonly occur among the Black African-Caribbean community while only seven (10.57%) felt that the problem was confined to the Bangladeshi community.

The explanation for Bangladeshis using drugs-included peer pressure, socialization, “it is fashionable,” curiosity, boredom, living in a different country (meaning here in the UK), family problems, hanging round bad people and using drugs while in Bangladesh, presumably referring to heroin which is available there at a fraction of the cost here in the UK.

Questions for an interview schedule were derived [Table 5] and three people agreed to interview. The semi-structured interviews gave insight into the understanding of drug problems among the Bangladeshi community in Leicester and confirmed the qualitative observations.

**Table 5:** Questions in the semi-structured interview schedule

1.	Is there a drug problem among the Bangladeshi community in Leicester?
2.	What drugs do you think are most commonly used?
3.	Why do you think Bangladeshis use drugs?
4.	What sort of impact is this having on families/community as a whole?
5.	Why do Asian families/communities find it difficult to talk about drugs/drug-related problems?
6.	What do you think families do or how do they react once they discover someone in the household is using drugs?
7.	Are there any real differences between Bangladeshis and other ethnic groups with regards to drug use?
8.	What do you feel should be done to improve local mainstream services?

## DISCUSSION

The literature review confirmed that there are known to be drug problems locally and nationally. People were willing to confirm that a drug problem existed yet they were hesitant to take part in the study formally even though anonymity was assured at all times. This questions whether conventional western research tactics are effective when exploring issues within different cultures. Perhaps it is now time to consider alternative and more effective ways of exploring sensitive issues among BME groups.

The gender differences in perceptions of drug use are not surprising. The acknowledgment in the small qualitative data that problem of drug abuse exists among Bangladeshis and the age, socialization, peer pressure, and ease of obtaining drugs are recognized as important factors. It is interesting that no mention was made of “alienation” or culture conflict as potential contributor which must be explored in future studies.

The gender differences as noted earlier by White[17] and Fountain *et al.*[5] are confirmed here. Social environments as identified by White[17] were also confirmed by our findings as was the early age use of cannabis. These similarities indicate that in spite of methodologic problems the findings are strikingly similar indicating that there is a recognizable drug problem in the community.

There are several limitations of the study which are important to bear in mind. The sample response was less than adequate and there were more males in the sample, but this may not be problematic because it is known to be males who are more likely to use drugs. The survey was carried out at one point in time, hence the results only reflect the opinions of a small section of the Bangladeshi community in Leicester. It is questionable how many females actually completed the questionnaires themselves. Inclusion of local schools and colleges may have provided a different picture and a greater cross-sectional representation. The problem of poor response and sample selection reflects problems of research in ethnic minorities. There is a need to go beyond just looking at the poor response rate and examine why this occurred in the first place. The topic itself may have prevented people from talking about a subject, which is sensitive and illegal. People may be frightened to talk openly especially if they are not fully sure as to what will happen to the information afterward and especially if it is seen as being collected “officially.” Fear of being identified as a “grass” and giving the community a bad name can have devastating consequences for individuals and their families by way of revenge and retaliation. As questionnaires were circulated through the mosque those who were more religious may have felt more pressure than those who were not religious at all or did not attend mosque. Better response rates may have been possible if other methods were used.

The present study should be seen as a pilot exploratory study to see what views a Bangladeshi community holds. The future research must build on these findings. The role of cultural values, cultural conflict, and acculturation must be explored further and links with religious factors understood.

## CONCLUSIONS

This study overtly confirms the existing knowledge of drug use in Bangladeshi community. Patterns that have been found elsewhere are also repeated here in Leicester. Drug problems exist in both males and females and among all age groups. There was a poor response rate to a formal survey, in spite of the fact that people are concerned about drug problems and its impact on individuals, family and the wider community. The results indicate that drug-related problems exist among the Bangladeshi community in Leicester. The biggest problem appears to be among the younger age group. Further work is urgently needed so that suitable culturally appropriate intervention packages can be developed.
